# FDA Acceptance of Surrogate End Points for Cancer Drug Approval: 1992-2019

**DOI:** 10.1001/jamainternmed.2020.1097

**Published:** 2020-04-27

**Authors:** Emerson Y. Chen, Alyson Haslam, Vinay Prasad

**Affiliations:** 1 Knight Cancer Institute, Division of Hematology and Medical Oncology, Oregon Health & Science University, Portland; 2 Department of Public Health and Preventive Medicine, Oregon Health & Science University, Portland

## Abstract

This retrospective review assesses the frequency of surrogate measures used for the first time vs subsequent times in a cancer setting and the surrogate’s strength of correlation with patient-centered outcomes.

The US Food and Drug Administration (FDA) approves cancer drugs based on direct measures of patient benefit—such as overall survival (OS) or quality of life—or surrogate measures, such as change in biomarker level or tumor size on imaging studies. Surrogate end points often have weak or unknown correlations with OS,^1,2^ and postmarketing studies are limited.^3^ A surrogate end point can be used repeatedly in a particular cancer setting, such as response rate (tumor shrinkage) in mantle cell lymphoma in 2013 (with ibrutinib and lenalidomide) and 2017 (with acalabrutinib) after it was accepted for the first time in 2006 (with bortezomib).^4^

One open question is how often does the FDA approve a drug based on a surrogate end point that has never been used before in treating that type of cancer? In this study, we assess the frequency of surrogate measures used for the first time vs subsequent times in a cancer setting and the surrogate’s strength of correlation with patient-centered outcomes.

## Methods

A retrospective review of cancer drugs approved from January 1992 through July 2019 on the basis of surrogate end points, either response rate or progression-free survival, was conducted using the FDA website and a previous systematic review.^5^ Data related to the drug, cancer type, approval basis, dates of approval, and postmarketing follow-up were extracted from FDA review documents, package inserts, or publications from PubMed (search terms were the drug name and cancer type).

The primary outcome measure was the determination of whether the surrogate end point was used for the first time or subsequent times in treatment of that cancer type. If drug A (eg, bortezomib in 2006) is the first drug approved for the treatment of a particular cancer type (eg, mantle cell lymphoma), based on response rate, followed by drugs B and C (eg, ibrutinib and lenalidomide in 2013),^4^ also based on response rate, then A is considered to be first and B and C are subsequent. We did not count molecular subtypes as separate cancer types, and breast cancer was the only form that was separated into localized and metastatic types. The strength of correlation between the surrogate end point and OS in that cancer type was based on the results of previous studies.^6^ Approval dates were plotted to describe the timing of accelerated and regular approvals based on first or subsequent surrogate end points.

## Results

We identified 194 unique drug authorizations for 132 drugs that were based on surrogate end points from 1992 through 2019. There were 89 accelerated approvals and 105 regular approvals (Table). A surrogate end point was used for the first time for a specific cancer type in 64 of 194 approvals (32.9%), and the remaining 130 of 194 (67.0%) were subsequent uses of the surrogate. The number of unique surrogate measure–cancer combinations accepted for drug approval has increased over time between 1992 and 2019 (Figure), to as many as 70 (36.1%) from 2016 through 2019 (Table). Most of these combinations had unknown postmarketing OS data (Table).

**Table,:** Characteristics of Cancer Drugs Approved on the Basis of Surrogate End Points, 1992-2019

Characteristic	FDA approval type, No. (%)	
	Accelerated	Regular
No. of drugs	194	
FDA approval status	89 (45.8)	105 (54.1)
Converted to regular	50 (25.7)	NA
Accelerated only	37 (19.1)	NA
Withdrawn from market	2 (1.0)	NA
Basis for approval (end points)		
Response rate	80 (41.2)	47 (24.2)
Progression-free survival	9 (4.6)	58 (29.9)
First vs repeated approvals		
First-surrogate based	28 (14.4)	36 (18.5)
Repeated	61 (31.4)	69 (35.6)
Period		
1992-1995	0	2 (1.0)
1996-1999	6 (3.1)	6 (3.1)
2000-2003	9 (4.6)	7 (3.6)
2004-2007	9 (4.6)	18 (9.3)
2008-2011	8 (4.1)	11 (5.7)
2012-2015	23 (11.8)	25 (12.9)
2016-2019	34 (17.5)	36 (18.6)
Postmarketing efficacy requirement		
OS or PRO not reported	58 (29.9)	55 (28.4)
OS or PRO known	31 (16.0)	50 (25.7)

Abbreviations: FDA, US Food and Drug Administration; NA, not applicable; OS, overall survival; PRO, patient-reported outcome.

**Figure.**
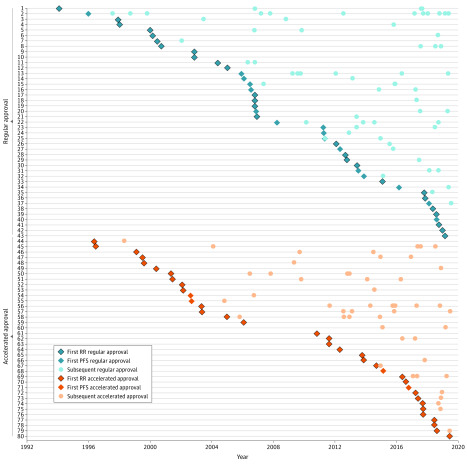
First-Surrogate–Based Regular and Accelerated Cancer Drug Approvals and Subsequent Use, 1992-2019 PFS indicates progression-free survival; RR, response rate; the listed numbers represent the following: 1, acute lymphoblastic leukemia (RR); 2, metastatic breast cancer (PFS); 3, indolent b-cell lymphoma (RR); 4, melanoma (RR); 5, T-cell lymphoma (RR); 6, hairy cell leukemia (RR); 7, prostate cancer (RR); 8, acute myeloid leukemia (RR); 9, gastric cancer (RR); 10, pancreatic cancer (RR); 11, myelodysplastic syndrome (RR); 12, metastatic breast cancer (RR); 13, kidney cancer (PFS); 14, gastrointestinal stromal tumor (PFS); 15, multiple myeloma (PFS); 16, ovarian cancer (PFS); 17, dermatofibroma protuberans (RR); 18, systemic mastocytosis (RR); 19, chronic eosinophilic leukemia (RR); 20, localized breast cancer (PFS); 21, mantle cell lymphoma (RR); 22, chronic lymphocytic leukemia (PFS); 23, melanoma (PFS); 24, medullary thyroid cancer (PFS); 25, neuroendocrine tumor (PFS); 26, basal cell carcinoma (RR); 27, soft tissue sarcoma (PFS); 28, chronic myelogenous leukemia (RR); 29, non–small cell lung cancer (RR); 30, giant cell tumor of bone (RR); 31, non–small cell lung cancer (PFS); 32, differentiated thyroid cancer (PFS); 33, lymphoplasmacytic lymphoma (RR); 34, follicular lymphoma (PFS); 35, diffuse large B-cell lymphoma (RR); 36, Erdheim-Chester disease (RR); 37, prostate cancer (PFS); 38, anaplastic thyroid cancer (RR); 39, pheochromocytoma (RR); 40, T-cell lymphoma (PFS); 41, squamous cell carcinoma (RR); 42, neurotrophic tyrosine kinase receptor fusion solid tumor (RR); 43, localized breast cancer (RR); 44, metastatic breast cancer (RR); 45, colorectal cancer (RR); 46, T-cell lymphoma (RR); 47, ovarian cancer (RR); 48, brain cancer (RR); 49, acute myeloid leukemia (RR); 50, chronic myelogenous leukemia (RR); 51, chronic lymphocytic leukemia (RR); 52, gastrointestinal stromal tumor (RR); 53, indolent B-cell lymphoma (RR); 54, colorectal cancer (PFS); 55, localized breast cancer (PFS); 56, non–small cell lung cancer (RR); 57, multiple myeloma (RR); 58, pheochromocytoma (RR); 59, kidney cancer (RR); 60, metastatic breast cancer (PFS); 61, tuberous sclerosis: giant cell astrocytoma (RR); 62, Hodgkin lymphoma (RR); 63, anaplastic large cell lymphoma (RR); 64, tuberous sclerosis: renal angiomyolipoma (RR); 65, localized breast cancer (RR); 66, mantle cell lymphoma (RR); 67, melanoma (RR); 68, multiple myeloma (PFS); 69, urothelial cancer (RR); 70, head and neck squamous cell carcinoma (RR); 71, soft tissue sarcoma (PFS); 72, Merkel cell carcinoma (RR); 73, microsatellite instability-high solid tumor (RR); 74, follicular lymphoma (RR); 75, hepatocellular carcinoma (RR); 76, gastric cancer (RR); 77, cervical cancer (RR); 78, mediastinal B-cell lymphoma (RR); 79, small cell lung cancer (RR); 80, diffuse large B-cell lymphoma (RR).

When examining the strength of correlation between the surrogate end point and OS among the 64 first-surrogate–based approvals, we found that 39 (61%) had no documented correlation, 10 (16%) had a poor correlation (*r* ≤ 0.7), 1 (2%) had a medium correlation (0.70 < *r* <0.85), and only 3 (5%) had a high correlation (*r* ≥ 0.85). Eleven approvals (17%) had varied levels of correlation across multiple validation studies. Among the 49 approvals with low or unknown correlation, 23 (47%) were accelerated approvals, and 26 (53%) were regular approvals.

## Discussion

The FDA has used surrogate end points approximately 194 times to approve cancer drugs since 1992, and about 1 in 3 times, a surrogate was used for the first time in a particular type of cancer. When this is the case, the strength of association between the surrogate end point and OS is often absent or weak. This means that the FDA’s use of these surrogate measures is justified neither by strength of association (ie, ability to predict gains in OS) nor previous first use, since 1 in 3 approvals constitute the use of a surrogate end point for the first time in the treament of a specific cancer type. Our study is limited by some missing FDA label updates before 2006 that are not publicly accessible.

Surrogate end points can expedite trial completion compared with OS,^4^ but they add substantial uncertainty regarding whether the drugs involved improve quantity or quality of life.^6^ Moreover, the FDA rarely demands stringent confirmation of clinical benefit following market approval.^3^ We find that the FDA is steadily accepting more surrogate measures over time, which are not justified by scientific validity or adherence to regulatory precedent. This reflects a greater tolerance of risk, and if postmarketing studies are slow, incomplete, or demonstrate negative results, then patients experience harm and cost without the intended benefit.
